# Dispersion of Multi-Walled Carbon Nanotubes Stabilized by Humic Acid in Sustainable Cement Composites

**DOI:** 10.3390/nano8100858

**Published:** 2018-10-20

**Authors:** Yuan Gao, Hongwen Jing, Mingrui Du, Weiqiang Chen

**Affiliations:** State Key Laboratory for Geomechanics & Deep Underground Engineering, China University of Mining & Technology, Xuzhou 221116, China; gaoyuan16@cumt.edu.cn (Y.G.); dumingruicumt@sina.com (M.D.); ts16030036a3@cumt.edu.cn (W.C.)

**Keywords:** humic acid (HA), MWCNT dispersion, stabilization, alkaline environment, cement

## Abstract

Multi-walled carbon nanotubes (MWCNTs) are promising nanoreinforcing materials for cement-based composites due to their superior material properties. Dispersion of MWCNTs is key for achieving the most effective way of enhancing efficiency, which is challenging in an alkaline cementitious environment. In this study, humic acid (HA) was used to stabilize the degree of dispersion of MWCNTs in an alkaline environment. The efficiency of HA in stabilizing MWCNT dispersion in cement composites was characterized using an ultraviolet spectrophotometer. The influences of HA on the workability and mechanical properties of ordinary Portland cement (OPC) reinforced with MWCNTs were evaluated, and the results revealed that the addition of HA can improve the stability of MWCNT dispersion in an alkaline environment. A concentration of 0.12 wt.% HA/S added to MWCNT suspensions was found to perform the best for improving the dispersion of MWCNTs. The addition of HA results in a decreased workability of the OPC pastes but has little influence on the strength performance. HA can affect the mechanical properties of OPC reinforced with MWCNTs by influencing the dispersion degree of the MWCNTs. An optimum range of HA (0.05–0.10 wt.%) is required to achieve the optimum reinforcing efficiency of MWCNTs.

## 1. Introduction

Due to their high aspect ratio, low density [1,2], and superior mechanical [3], thermal [4], and electrical properties [5], multi-walled carbon nanotubes (MWCNTs) have proven to be quite efficient in reinforcing the material properties of organic polymers [6], biomaterials [7], and ceramics [8]. Recent studies [9,10,11,12] have shown that the superior material properties of MWCNTs make them promising candidates to be mixed into ordinary Portland cement (OPC) for the purpose of strength improvement and microstructure reinforcement. Zou et al. [13] added 0.075 wt.% of MWCNTs into OPC pastes and improved the flexural strength and elastic modulus by 63% and 32%, respectively. Xu et al. [14] determined that MWCNTs can fill nano-scale gel pores between calcium silicate hydrate products and reduce the porosity of OPC pastes.

In order to achieve the maximum enhancing efficiency, MWCNTs should be dispersed individually in the final matrices [15]. Agglomerated MWCNTs function as defects that lead to stress concentration, resulting in the degradation of strength performance [9,13]. Pristine MWCNTs interact strongly with each other due to the van der Waals attraction [16,17] and tend to form large agglomerates. Currently, MWCNT suspensions are generally used as alternatives to pristine MWCNTs for addition to matrices [18]. MWCNT suspensions are prepared by adding surfactants such as air entrainers, calcium naphthlaene sulfonate, and polycarboxylates under ultrasonication [18]. However, the stability of dispersed MWCNTs in aqueous solutions is challenging to achieve in the alkaline environment of cementitious mixtures [19]. In cementitious mixtures, surfactants will chemically react with ions (i.e., Ca^2+^, Na^+^, K^+^, SO^4−^, and OH^−^ ions) [20,21], thus the amount of surfactant molecules participating in the dispersion of MWCNTs will decrease, resulting in re-agglomeration of the MWCNTs. In previous studies [19], it has been found that re-agglomerated MWCNTs in fresh cementitious mixtures can reach over 80% after 18 h. Du et al. [22] have found that methylcellulose can help to stabilize dispersed MWCNTs in an alkaline environment owing to the viscosity-increasing effect that retards the movement of MWCNTs. However, the in-situ applications of the prepared cementitious mixtures will be limited owing to the decreased workability, especially in conditions where high fluidity is required [15,23]. Workability is another significant factor for fresh OPC pastes in civil engineering, particularly grouting projects [23], which has a major influence on the transport of cement particles when the mixtures are fresh. Maintaining a high water-to-cement (*w*/*c*) ratio has some benefits, such as increasing workability and reducing the carbon footprint [22]. Although lowering the *w*/*c* ratio is one of the most common methods to increase the strength of cement pastes, benefits such as workability will suffer severely. Therefore, the introduction of well-dispersed MWCNTs into cementitious composites holds the potential to overcome the shortage of paste strength.

Humic acid (HA), a biological macromolecule, is one of the most common natural organic matters. It is neutral in aqueous solutions, environmentally-friendly, and non-toxic [24]. The mass content of hydroxy and phenolic hydroxyl functional groups in HA is generally lower than 1% [25]. Strong steric repulsion forces exist between HA molecules [26]. Previous studies [27,28] have found that HA can absorb onto the surface of MWCNTs through electrostatic interaction, π-π interaction, hydrophobic interaction, and hydrogen bonding, and the dispersion of MWCNTs in water can be stabilized due to the steric repulsion effect. Han et al. [26] have found that the addition of 0.005 wt.% HA to 0.01 wt.% carbon black nanoparticles can increase the dispersion degree of carbon black nanoparticles in an aquatic environment by 78%. Saleh et al. [25] have found that mixing 0.002 wt.% HA in aqueous solutions could improve the colloidal stability of MWCNTs and reduce the re-aggregation rate by about two orders of magnitude. In work conducted by Yang et al. [29], HA was found to be quite effective in enhancing the dispersibility of nanobiochar in an alkaline aqueous medium with pH higher than 10. Since the steric repulsion effect of HA is not influenced by the alkaline environment [29] and the mass content of functional groups is so low that chemical reactions between HA and cement will not happen [24], HA holds the potential to be used to stabilize the dispersion of MWCNTs in cementitious composites.

In this paper, the stabilizing effect of HA on the dispersion of MWCNTs in OPC was experimentally studied. The dispersion degree of MWCNTs in simulated cementitious pore solutions and aqueous solutions containing HA was characterized using ultraviolet-visible spectrophotometry (UV-vis). The workability and mechanical properties of the MWCNT-enhanced OPC matrices were studied. The nanostructure of MWCNT-reinforced OPC matrices was investigated using a scanning electron microscope (SEM). The results of this study reveal that the addition of HA can improve the stability of MWCNT dispersion in an alkaline environment, with an increase of 21.9–45.8% compared to no HA. An optimum range of HA (0.05–0.10 wt.%) can achieve the best enhancement efficiency of the mechanical performance of OPC reinforced with MWCNTs. The findings of this study provide a new method for improving the degree of dispersion of MWCNTs and guide further understanding of MWCNT-OPC composites.

## 2. Experimental Process

### 2.1. Materials and Instrumentation

OPC, type P.O. 42.5, referring to the requirements of Chinese Standard GB175-2007 [30], was used as the binder material in this study. The commercially purchased MWCNTs have an average diameter of 5–15 nm and an average length of 10–20 µm. A commercial polycarboxylate-based surfactant (PC) was used as the dispersing agent to improve the dispersion degree of the MWCNTs in water [13]. HA containing two types of functional groups (i.e., hydroxy and phenolic hydroxyl) with a mass content of 0.9% was used.

A horn ultrasonicator (VCX 500W) (SONICS Inc., Newtown, CT, USA) with a 13-mm-diameter cylindrical tip was used to disperse the agglomerated MWCNTs in an aqueous solution.

UV-vis tests were performed using ultraviolet-visible spectrophotometry (TU-1901) (PERSEE Inc., Beijing, China) to characterize the dispersion degree of the MWCNTs.

The uniaxial compression and flexural tests of the specimens were carried out using a DNS100 electronic universal testing machine (Changchun research institute for mechanical science Co., Ltd, Changchun, China).

A scanning electron microscope (SEM, Su8200) (HITACHI Inc., Tokyo, Japan) was used to investigate the microstructure of the hardened MWCNT-OPC pastes.

### 2.2. Preparation of MWCNT Suspensions

**Figure 1:**
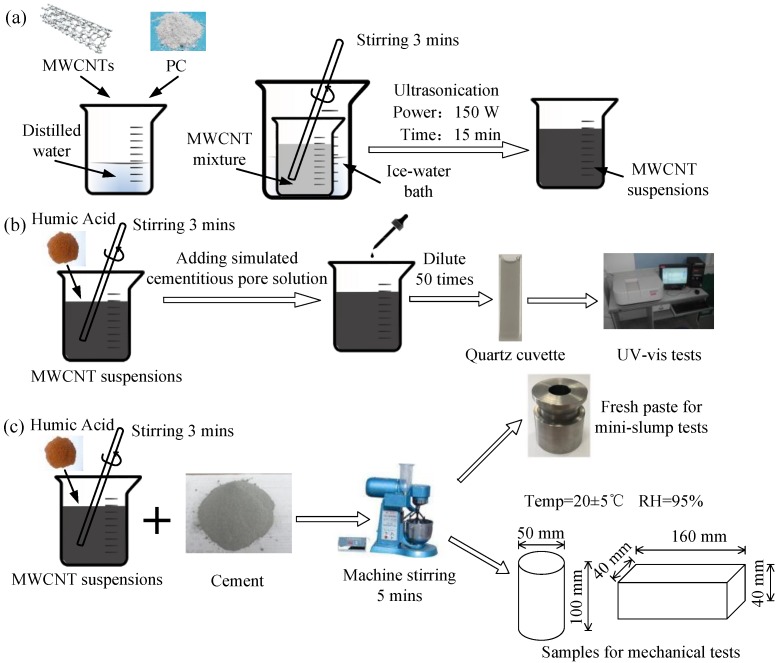
Preparation of: (**a**) Multi-walled carbon nanotubes (MWCNT) suspensions; (**b**) MWCNT samples for UV tests; (**c**) MWCNT-OPC samples for mini-slump tests and the characterization of mechanical properties.

The suspensions were prepared by mixing MWCNT powder with PC in distilled water under ultrasonication (Figure 1a). The ultrasonication power and time were fixed at 150 W and 15 min, respectively. To prevent overheating, the suspensions were placed in an ice-water bath. According to previous studies [13,19,22], the concentration of the MWCNT powder was defined as 0.08 wt.% relative to the weight of the suspensions. The PC-to-suspension ratio was 0.64 wt.%, which was eight times the concentration of the MWCNTs.

As shown in Figure 1b, the MWCNT suspensions were first subjected to ultrasonication before adding the HA powder and stirring for 3 minutes [31]. The concentration of HA was defined by the weight ratio of the HA powder to the MWCNT suspension (HA/S), and four concentrations of 0.00 wt.%, 0.12 wt.%, 0.25 wt.%, and 0.50 wt.% were designed, as exhibited in Table 1. Both the preparation of the MWCNT suspensions and the dissolving of the HA powder were conducted at room temperature (20 ± 5 °C). To test the stabilization of the MWCNT suspensions in an alkaline environment, a simulated cementitious pore solution was added into the suspensions after ultrasonication. The concentrations of chemicals [21] in the simulated OPC pore solution are presented in Table 2. The composition of the cementitious pore solution provided the Ca^2+^, Na^+^, K^+^, SO^4−^, and OH^−^ ions found in early-age (within 8 h) concrete [20].

### 2.3. Preparation of Specimens

The specimens were cast in steel molds (50 mm × 100 mm and 40 mm × 40 mm × 160 mm) after mixing each solution (containing 0.00 wt.%, 0.05 wt.%, 0.10 wt.%, and 0.20 wt.% of HA powder) and cement (using HA/G to represent the weight ratio of HA to cement; see Table 1). Then, the solutions and the cement powder were mixed based on a water-to-cement ratio (*w*/*c*) of 0.4 (Figure 1b). Two types of specimens were prepared for the compressive and flexural strength tests. The samples were demolded after 24 h and cured in a saturated lime water bath at a temperature of 20 ± 5 °C and a relative humidity of 95% for another 27 d before testing [22]. Aqueous solutions containing distilled water, PC, and HA powder were used to make the reference cementitious pastes (Ref pastes; see Table 1).

### 2.4. Characterization

#### 2.4.1. Dispersion Tests for MWCNT Suspensions

The time-dependent stabilization of the prepared MWCNT suspensions in alkaline environments was characterized by UV-vis. The measured absorbance (ABS) at a certain wavelength can reflect the degree of dispersion [32,33]. The prepared solutions were diluted by a factor of 50 and tested at a wavelength (λ) of 500 nm every hour up to 18 h after mixing to calculate the dispersed MWCNT concentration (*C_d_*). Although the cement paste hardening normally happens at about 3–4 h, the initial setting time seriously delayed by higher *w*/*c* and the environmental humidity, particularly in the underground grouting engineering projects [23]. Therefore, in this study, the UV-vis tests measured 0–18 h to ensure the cement paste hardened, which is consisted with previous research studies [19,22]. Three samples were measured in each UV-vis test to ensure that the concentration of MWCNTs in the solution was consistent. *C*_d_ was calculated using Beer-Lambert’s law [34,35,36], given as:

(1) $$C_{d}=\frac{A}{\varepsilon l}$$

where *A* is the average absorbance, *ε* is the extinction coefficient [34,35,36], and *l* = 1 cm is the optical path length of the light through the MWCNT suspensions.

To determine *ε*, the well-dispersed suspensions with 0.08 wt.% MWCNTs were first diluted with distilled water by factors of 50, 75, 100, 125, and 150.

**Figure 2:**
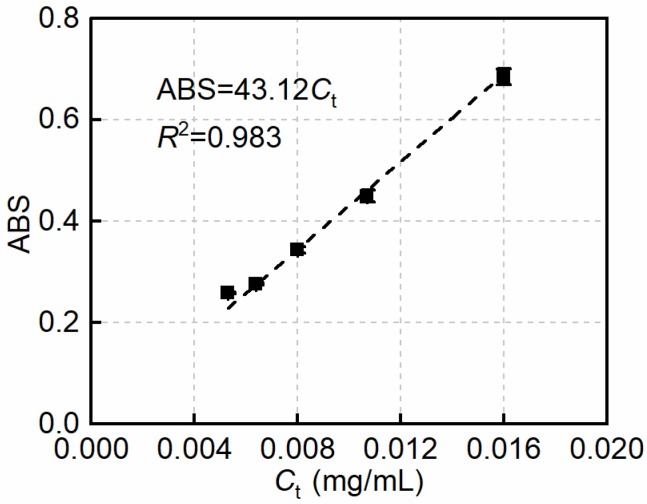
The zero-intercept linear relationship between absorbance (ABS) and *C*_t_ in solutions at λ = 500 nm.

The above suspensions were thoroughly ultrasonicated to guarantee that the maximum ABS was reached. *ε* was then determined by fitting a zero-intercept linear correlation between the measured ABS and the theoretical maximum concentration of the suspensions (*C*_t_). The zero-intercept linear regression equation in Figure 2 suggests that *ε* is 43.12 mL mg^−1^ cm^−1^, which is consistent with the reported values between 41 and 46 mL mg^−1^ cm^−1^ [34,35,36]. The goodness of fit is indicated by the correlation coefficient (*R*^2^) of the equation (98.3%).

#### 2.4.2. Characterization of Workability

**Figure 3:**
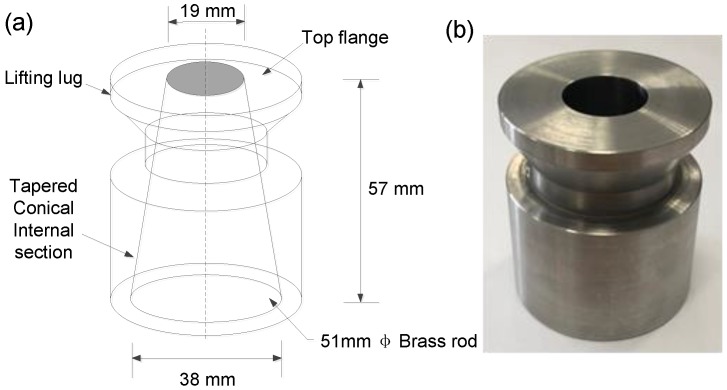
(**a**) Dimensions and (**b**) mini-cone for mini-slump test.

The workability of fresh MWCNT-OPC and plain OPC pastes was measured using mini-slump tests [18]. The setup and geometry of the mini-slump cone are presented in Figure 3. The cone was first placed on a flat sheet, then fresh paste was poured into the cone and compacted. The excess paste was then removed from the top surface, and the cone was lifted vertically to ensure minimal lateral disturbance during the tests. The diameter of the hardened spread samples was measured at five different locations around the outline after 24 h. Workability was defined as the average measurement.

#### 2.4.3. Mechanical Properties and Microstructure Tests

The compressive strength (*σ*_c_) of the MWCNT-OPC pastes were measured using a universal testing machine. Constant loading rates of 0.5 mm min^−1^ were adopted for the compressive strength tests. Three specimens were prepared and tested for each paste.

**Figure 4:**
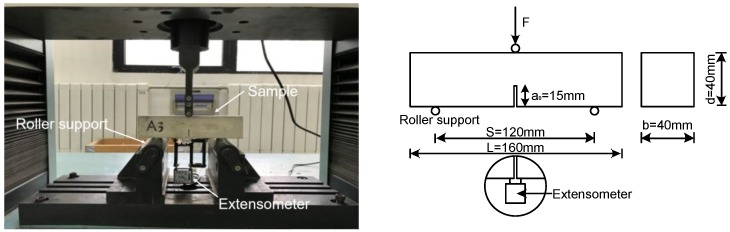
Three-point bending test setup and configuration.

The flexural strength (*σ*_t_), Young’s modulus (*E*), and fracture energy (*G*_F_) of the cement pastes were measured using three-point bending tests [13], as presented in Figure 4. An extensometer with a gauge length of 50 mm and a +5 mm measurement range was attached to the beam with a glue stick. A constant loading rate of 0.1 mm min^−1^ was adopted. Both the crack mouth opening displacement (CMOD) and the load line deflection were measured. Three specimens were prepared and tested for each paste.

Based on the area under the load–deflection curve, *G*_F_ can be calculated using the following equation [37,38]:

(2) $$G_{F}=\frac{A_{0}+mg\delta_{0}}{(d-a_{0})b}$$

where *A*_0_ is the area under the load–deflection curves, *mg* is the weight of the sample, *δ*_0_ is the deflection of the beam at final failure, *b* is the beam width, *d* is the depth, and *a*_0_ is the notch depth. *E* was calculated based on the initial linear elastic segment of the load–CMOD curves [37]. *σ*_t_ was calculated from the peak load results [39].

The microstructure of the fracture surface was characterized using SEM to observe the dispersion of the MWCNTs in the hardened pastes.

## 3. Results and Discussion

### 3.1. Stabilizing Effect of HA on the Dispersion of MWCNTs in an Alkaline Environment

**Figure 5:**
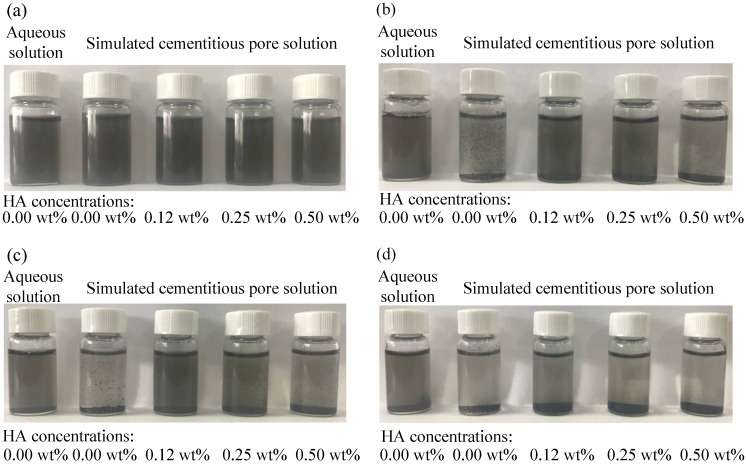
MWCNT suspensions containing different concentrations of humic acid (HA) in cementitious pore solutions and aqueous solution at different times: (**a**) 0 h; (**b**) 3 h; (**c**) 6 h; (**d**) 18 h after ultrasonication.

Figure 5 presents the MWCNT suspensions with different HA/S in an alkaline pore solution and an aqueous solution at different times after ultrasonication. Visual observations show that all the prepared suspensions possessed a uniform black color. Re-agglomeration and sedimentation of MWCNTs in the suspensions occurred after settling for 3 h, especially for the suspensions in the alkaline pore solution without HA. After 18 h, most of the MWCNTs settled to the bottom and the color of the aqueous solutions became gray with greater transparency. The suspensions in pore solutions with HA/S of 0.12 wt.% remained darker in color than the suspensions in alkaline pore solutions with other values of HA/S, indicating that more MWCNTs were in dispersion.

**Figure 6:**
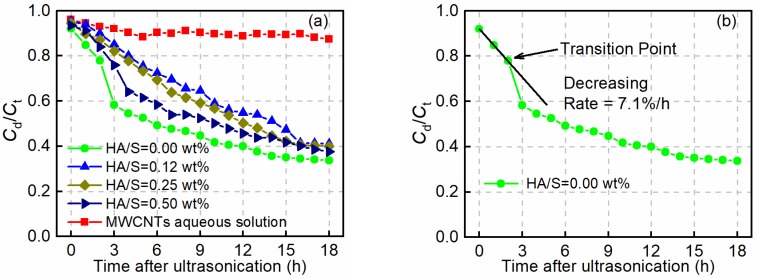
(**a**) Stabilization of MWCNT dispersion in different solutions after mixing; (**b**) determination of the transition point and decreasing rate of *C*_d_/*C*_t_. (Note: For clarity of presentation, the error bars are not presented.).

Figure 6a shows the calculated *C*_d_/*C*_t_ over time using Equation (1), based on UV-vis measurements. *C*_d_/*C*_t_ quantifies the degree of dispersion, and a higher ratio means more MWCNTs are in the dispersed state [19]. Figure 6a shows that the dispersion of MWCNTs in aqueous solution is very stable, with the *C*_d_/*C*_t_ decreasing by less than 9% after 18 h. In contrast, an obvious decline in *C*_d_/*C*_t_ is observed in other curves in Figure 6a, indicating that the addition of a simulated pore environment reduces the degree of MWCNT dispersion. Over time, more MWCNTs tend to re-agglomerate, and *C*_d_/*C*_t_ decreases constantly to less than 40% after 18 h. Before 15 h, however, suspensions with HA/S of 0.12 wt.% are found to maintain more dispersed MWCNTs, increasing the stability by 21.9–45.8% compared to no HA.

It is generally accepted that for cementitious mixtures with *w*/*c* of 0.4–0.6, the initial setting happens at about 3–4 h [19] and after that, the dispersion degree of MWCNTs will be less affected. At 3 h, the dispersion degree of MWCNT suspensions containing 0.12 wt.% HA is about 85%, whereas that of MWCNT suspensions without HA is about 58%. When HA/S exceeds 0.12 wt.%, the dispersion degree declines by 89–97% compared to that of suspensions with HA/S of 0.12 wt.%, indicating that an optimum mass concentration range of HA is required for achieving the maximum stabilizing effect. Because HA is a biomacromolecule and will absorb onto the surface of MWCNTs at a low concentration, which contributes to the increment of the steric repulsion in suspensions [40], the van der Waals forces will decrease and consequently delay the re-aggregation of MWCNTs [41,42]. However, when the HA/S exceeds 0.12 wt.%, more adsorption of HA becomes less effectual for dispersion due to HA having a moderate molecular weight (308.24 g/mol) and containing abundant functional groups [43] that might link adjacent MWCNTs into large agglomerations [44,45].

Figure 6b shows a transition point during the decline of *C*_d_/*C*_t_, which can be identified by the curve’s gradient. As shown in Figure 6b, before the transition point, the decrease of *C*_d_/*C*_t_ can be regarded as linear (7.1% per hour for MWCNT suspensions without HA), whereas after the transition point, *C*_d_/*C*_t_ experiences its maximum decrease in the 18 h period. The corresponding time for each transition point is measured for all curves (except MWCNTs in aqueous solution) in Figure 6a and listed in Table 3. Decreasing rates of *C*_d_/*C*_t_ are also found using linear regression (Table 3, column 3). The rate of decrease is affected by the HA concentration, and a moderate concentration of HA enhances the steric repulsion and stability of suspensions in an alkaline environment [46,47]. Furthermore, the transition points are obviously delayed by adding HA, especially at 0.12 wt.% HA concentration, which had a transition point at 9 h.

### 3.2. Effect of HA on the Workability of Fresh MWCNT-OPC Pastes

**Figure 7:**
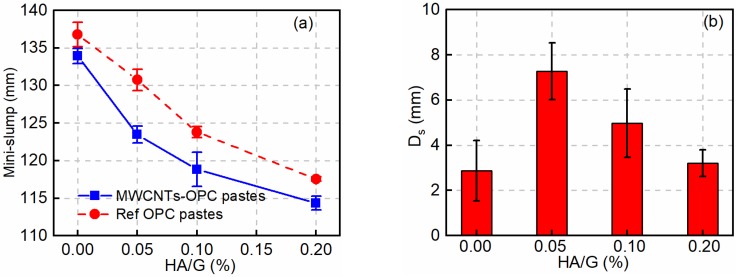
(**a**) Mini-slump spreads of fresh MWCNT-OPC and plain OPC pastes; (**b**) deterioration of slump diameter (*D*_s_) caused by MWCNTs at different HA/G.

The mini-slump spread versus HA/G is shown in Figure 7a, from which it can be seen that the spread diameter of plain or MWCNT-OPC pastes decreases gradually as HA/G increases from 0.00 wt.% to 0.20 wt.%. For the plain OPC pastes, the slump diameter declined to 117.6 mm from 136.8 mm when 0.20 wt.% of HA was added. The adverse effect of HA on the workability of OPC is due to the adsorption between HA and PC becoming kinetically less favorable [48], resulting in electrostatic repulsion and elevated steric [22] hindrance between the collector surface and HA, thus inhibiting the workability of fresh pastes.

As can be seen from Figure 7a, adding MWCNTs to the OPC pastes leads to a more significant decrease in the mini-slump diameter. The further deterioration in workability is affected by two factors: The first factor is that MWCNTs can act as nucleation sites to accelerate the hydration reaction and therefore decrease the workability of pastes [49]; the second factor is that the dispersed MWCNTs can absorb PC molecules [18], resulting in a decreased amount of PC interacting with cement powders in the MWCNT pastes than in the Ref pastes. Figure 7b shows the deterioration of slump diameter (*D*_s_) influenced by mixing MWCNTs at the same concentration of HA/G. The maximum deterioration of the slump diameter at 0.05 wt.% HA/G is 7.2 mm, decreasing from 130.7 mm for the Ref paste to 123.5 mm for the MWCNT-OPC paste. This is likely due to more MWCNTs being in a dispersed state at 0.12 wt.% HA/S (discussed in Section 3.1), accelerating a stronger hydration reaction and absorbing more PC molecules.

### 3.3. Effect of HA on the Mechanical Properties of MWCNT-OPC Pastes

**Figure 8:**
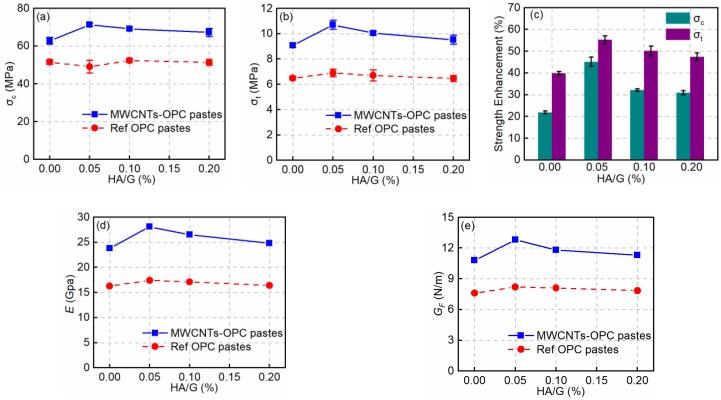
Variations of: (**a**) *σ*_c_ and (**b**) *σ*_t_ of cement pastes at the age of 28 days versus concentration of HA; (**c**) Strength enhancement percent versus concentration of HA; (**d**) *E* and (**e**) *G_F_* of cement pastes at the age of 28 d versus concentration of HA. (Note: Due to the error bars in Figure **d**,**e** being too small, for clarity of presentation they are not presented).

The average values of the compressive strength (*σ*_c_) and flexural strength (*σ*_t_) and their variations versus HA/G are presented in Figure 8. *σ*_c_ and *σ*_t_ of Ref pastes without any MWCNTs vary within the range of 49.1–52.3 MPa and 6.5–6.9 MPa, respectively, with a maximum variation of about 6%. This observation indicates that the addition of HA contributes little to the ultimate strength performance of cement pastes.

After adding the MWCNTs to the OPC pastes, *σ*_c_ and *σ*_t_ of all samples increased. Since both the addition of PC [13] and HA makes little difference to the ultimate strength of cement matrices when the *w*/*c* is constant, the improved mechanical properties of the hardened pastes are mainly due to the addition of MWCNTs. For MWCNT-OPC pastes, *σ*_c_ ranges from 62.8 to 71.2 MPa and *σ*_t_ from 9.1 to 10.7 MPa, increasing by about 22–45% and 40–55%, respectively, when HA was added. As can be seen from Figure 8d,e, the overall variations of *E* and *G*_F_ against HA/G are correlated. It is found that the *E* and *G*_F_ increase with mixing HA. The optimal ranges of HA/G for improving ductility and fracture energy are found to be 0.05–0.10. Compared with the control sample without HA, the maximum enhancement achieved with HA is found at 0.05, where *E* and *G*_F_ are improved by about 56.1–61.5%.

Figure 8c shows that the maximum increment of strength enhancement was achieved at an HA/G of 0.05 wt.%. The maximum increments of *σ*_c_ and *σ*_t_ are about 45% and 55%, respectively. With more HA added to the OPC pastes, the enhancement effect is lower than that of the optimal concentration range. This finding is in accordance with the dispersion degree of the MWCNTs shown in Figure 8a. When there is no HA (HA/G = 0.00 wt.%) or too much HA (HA/G = 0.20 wt.%), the stable dispersion of MWCNTs in an alkaline environment is decreased. Therefore, the enhancing efficiency of MWCNTs on *σ*_c_ and *σ*_t_ becomes poorer when HA/G is 0.00 wt.% or 0.20 wt.%. An appropriate amount of HA (0.05 wt.% to 0.10 wt.%) is beneficial for the uniform dispersion of MWCNTs (Figure 6) and correspondingly enhances the *σ*_c_ and *σ*_t_ of the specimens (Figure 8).

### 3.4. Distribution of MWCNTs in the OPC Matrix

In addition to mechanical performance reinforcement, MWCNTs can also influence the microstructure of the cement pastes, which is also linked with the long-term performance of the composite materials [50,51]. Previous research has suggested that MWCNTs can fill nano/micro-sized pores [15,52]. Nanomaterials may fill gel pores between calcium silicate hydrate products, contributing to a reduction in the porosity and an increment in stiffness [15,52,53]. On the other hand, MWCNTs can bridge capillary pores and enhance higher load capacity, ductility, and fracture energy of the cement pastes [53]. However, the agglomeration of MWCNTs may heavily decrease the function of nanomaterials and degrade strength performance [9,13]. To better investigate the reinforcing potential of HA benefiting the dispersion stabilization of MWCNTs in cement composites, the fracture surface of the sample was observed under SEM.

**Figure 9:**
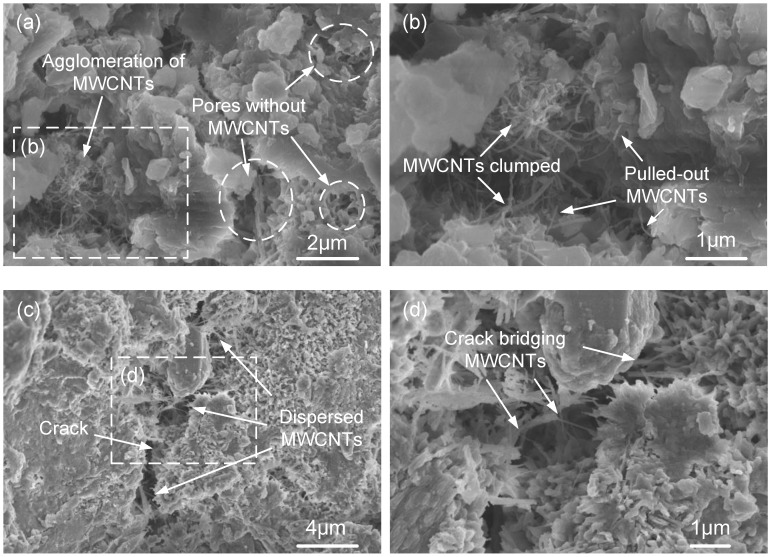
Scanning electron microscope (SEM) images of MWCNTs in the cement matrix with: (**a**,**b**) HA/G = 0.00 wt.%; (**c**,**d**) HA/G = 0.05 wt.%.

As presented in Figure 9a, the agglomeration of MWCNTs appears around the hydration products in one pore, while there are hardly any MWCNTs visible in other pores. The observation of the aggregation of MWCNTs is consistent with the measured *C*_d_/*C*_t_ (Figure 6a) in Section 3.1. Furthermore, the agglomerations are likely the cause of the clumped MWCNTs and “pulled-out” MWCNTs shown in Figure 9b, which are in turn the cause of the decreased mechanical properties, as presented in Section 3.3 [13].

Figure 9c,d show that the observable agglomerations of MWCNTs are markedly reduced at 0.05 wt.% HA/G. Several cracks can be seen on the surface of the cement paste where an appropriate amount of dispersed MWCNTs is exposed. Moreover, a large amount of MWCNTs with sufficient length can bridge capillary cracks or pores (at the nano/micrometer scale) [52,53], indicating that the well-dispersed MWCNTs could act as a crack bridging material and have the capacity to inhibit crack propagation, therefore providing improved mechanical performance of the cement matrix, as discussed in Section 3.3.

This study was consistent with the dispersion stabilization measurements and with the viewpoints presented in previous research: more dispersed MWCNTs implies that the tube-paste interfacial bonding is stronger, possibly resulting from the covalent bonds formed between the crack bridging MWCNTs and the pastes [13,52]. The unique capability of HA suggests that the combination of HA and MWCNTs may have the potential to reinforce both the strength and the ductility of cement pastes.

## 4. Conclusions

This study investigated the effects of HA on the dispersion of MWCNT suspensions in an alkaline environment and the workability and mechanical properties of MWCNT-OPC pastes. The main findings are as follows:

(1) The addition of HA works effectively for stabilizing the dispersion degree of MWCNT suspensions. The addition of 0.12 wt.% HA gave the best performance in stabilizing the MWCNT dispersion in an alkaline environment, increasing the stability by 21.9–45.8%.

(2) The addition of HA decreases the workability of OPC pastes, whereas it has little influence on the mechanical properties of the hardened OPC. 

(3) HA can affect the mechanical performance of MWCNT-reinforced OPC pastes by influencing the dispersion degree of the MWCNTs. An appropriate range of the mass concentration of HA (0.05–0.10 wt.%) is required to achieve optimum enhancing efficiency of the MWCNTs. The maximum increments in the compressive strength, flexural strength, Young’s modulus, and fracture energy are 45%, 55%, 61% and 56%, respectively. 

(4) HA markedly improves the degree of dispersion of MWCNT suspensions by absorbing onto the surface of MWCNTs at a low concentration by enhancing the steric repulsion. The combination of HA may decrease the van der Waals forces among MWCNTs and inhibit the re-aggregation of MWCNTs. The SEM images show that the MWCNTs were distributed more uniformly in the cement matrices when HA was incorporated.

## Figures and Tables

**Table 1:** Mix design of plain ordinary Portland cement (OPC) and multi-walled carbon nanotube- ordinary Portland cement (MWCNT-OPC) pastes.

Mix	C/S (wt.%)	P/S (wt.%)	HA/S (wt.%)	C/G (wt.%)	P/G (wt.%)	HA/G (wt.%)
Ref	0.08	0.64	0	0.032	0.256	0
MWCNTs-1			0			0
MWCNTs-2			0.12			0.05
MWCNTs-3			0.25			0.10
MWCNTs-4			0.50			0.20

Note: C/S and P/S represent MWCNT- and PC-to-suspension weight percentages. C/G and P/G represent MWCNT- and PC-to-cement weight percentages.

**Table 2:** Concentration of prepared chemicals in the simulated cementitious pore solution.

Compounds	NaOH	KOH	CaSO_4_·2H_2_O	Ca(OH)_2_
Concentration (g/L)	8	22.4	27.6	Saturated

**Table 3:** Transition time in the dispersion state.

HA Concentration (wt.%)	Transition Point (h)	Rate of Decrease of *C*_d_/*C*_t_ (% per h)
0.00	2	7.1
0.12	9	3.4
0.25	6	4.2
0.50	3	5.9
